# Inhibition of ATR opposes glioblastoma invasion through disruption of cytoskeletal networks and integrin internalization via macropinocytosis

**DOI:** 10.1093/neuonc/noad210

**Published:** 2023-11-04

**Authors:** Sarah J Derby, Louise Dutton, Karen E Strathdee, Katrina Stevenson, Anna Koessinger, Mark Jackson, Yuling Tian, Wenxi Yu, Kathy Mclay, Josette Misquitta, Sama Alsharif, Cassie J Clarke, Lesley Gilmour, Peter Thomason, Ewan McGhee, Connor L McGarrity-Cottrell, Aurelie Vanderlinden, Spencer J Collis, Ola Rominyi, Leandro Lemgruber, Gergely Solecki, Michael Olson, Frank Winkler, Leo M Carlin, Dieter Henrik Heiland, Gareth J Inman, Anthony J Chalmers, Jim C Norman, Ross Carruthers, Joanna L Birch

**Affiliations:** Wolfson Wohl Translational Cancer Research Centre, School of Cancer Sciences, University of Glasgow, Glasgow, UK; Wolfson Wohl Translational Cancer Research Centre, School of Cancer Sciences, University of Glasgow, Glasgow, UK; Wolfson Wohl Translational Cancer Research Centre, School of Cancer Sciences, University of Glasgow, Glasgow, UK; Wolfson Wohl Translational Cancer Research Centre, School of Cancer Sciences, University of Glasgow, Glasgow, UK; Wolfson Wohl Translational Cancer Research Centre, School of Cancer Sciences, University of Glasgow, Glasgow, UK; CRUK Scotland Institute, Glasgow, UK; Wolfson Wohl Translational Cancer Research Centre, School of Cancer Sciences, University of Glasgow, Glasgow, UK; Wolfson Wohl Translational Cancer Research Centre, School of Cancer Sciences, University of Glasgow, Glasgow, UK; Wolfson Wohl Translational Cancer Research Centre, School of Cancer Sciences, University of Glasgow, Glasgow, UK; Wolfson Wohl Translational Cancer Research Centre, School of Cancer Sciences, University of Glasgow, Glasgow, UK; Wolfson Wohl Translational Cancer Research Centre, School of Cancer Sciences, University of Glasgow, Glasgow, UK; Wolfson Wohl Translational Cancer Research Centre, School of Cancer Sciences, University of Glasgow, Glasgow, UK; CRUK Scotland Institute, Glasgow, UK; Wolfson Wohl Translational Cancer Research Centre, School of Cancer Sciences, University of Glasgow, Glasgow, UK; CRUK Scotland Institute, Glasgow, UK; CRUK Scotland Institute, Glasgow, UK; Department of Oncology and Metabolism, The University of Sheffield Medical School, Sheffield, UK; Department of Oncology and Metabolism, The University of Sheffield Medical School, Sheffield, UK; Department of Oncology and Metabolism, The University of Sheffield Medical School, Sheffield, UK; Department of Oncology and Metabolism, The University of Sheffield Medical School, Sheffield, UK; Cellular Analysis Facility, College of Medical, Veterinary & Life Sciences, University of Glasgow, Glasgow, UK; German Cancer Consortium (DKTK), German Cancer Research Center (DKFZ), Heidelberg, Germany; Department of Chemistry and Biology, Ryeson University, Toronto, Ontario, Canada; German Cancer Consortium (DKTK), German Cancer Research Center (DKFZ), Heidelberg, Germany; Wolfson Wohl Translational Cancer Research Centre, School of Cancer Sciences, University of Glasgow, Glasgow, UK; CRUK Scotland Institute, Glasgow, UK; MILO Laboratory, Department of Neurosurgery, Freiburg, Germany; Wolfson Wohl Translational Cancer Research Centre, School of Cancer Sciences, University of Glasgow, Glasgow, UK; CRUK Scotland Institute, Glasgow, UK; Wolfson Wohl Translational Cancer Research Centre, School of Cancer Sciences, University of Glasgow, Glasgow, UK; Wolfson Wohl Translational Cancer Research Centre, School of Cancer Sciences, University of Glasgow, Glasgow, UK; CRUK Scotland Institute, Glasgow, UK; Wolfson Wohl Translational Cancer Research Centre, School of Cancer Sciences, University of Glasgow, Glasgow, UK; Wolfson Wohl Translational Cancer Research Centre, School of Cancer Sciences, University of Glasgow, Glasgow, UK

**Keywords:** ATR, DNA damage response, glioblastoma, high-grade glioma, invasion, macropinocytosis

## Abstract

**Background:**

Glioblastomas have highly infiltrative growth patterns that contribute to recurrence and poor survival. Despite infiltration being a critical therapeutic target, no clinically useful therapies exist that counter glioblastoma invasion. Here, we report that inhibition of ataxia telangiectasia and Rad 3 related kinase (ATR) reduces invasion of glioblastoma cells through dysregulation of cytoskeletal networks and subsequent integrin trafficking.

**Methods:**

Glioblastoma motility and invasion were assessed in vitro and in vivo in response to ATR inhibition (ATRi) and ATR overexpression using time-lapse microscopy, two orthotopic glioblastoma models, and intravital imaging. Disruption to cytoskeleton networks and endocytic processing were investigated via high-throughput, super-resolution and intravital imaging.

**Results:**

High ATR expression was associated with significantly poorer survival in clinical datasets while histological, protein expression, and spatial transcriptomics using glioblastoma tumor specimens revealed higher ATR expression at infiltrative margins. Pharmacological inhibition with two different compounds and RNAi targeting of ATR opposed the invasion of glioblastoma, whereas overexpression of ATR drove migration. Subsequent investigation revealed that cytoskeletal dysregulation reduced macropinocytotic internalization of integrins at growth-cone-like structures, resulting in a tumor microtube retraction defect. The biological relevance and translational potential of these findings were confirmed using two orthotopic in vivo models of glioblastoma and intravital imaging.

**Conclusions:**

We demonstrate a novel role for ATR in determining invasion in glioblastoma cells and propose that pharmacological targeting of ATR could have far-reaching clinical benefits beyond radiosensitization.

## Graphical Abstract

**Graphical Abstract:**
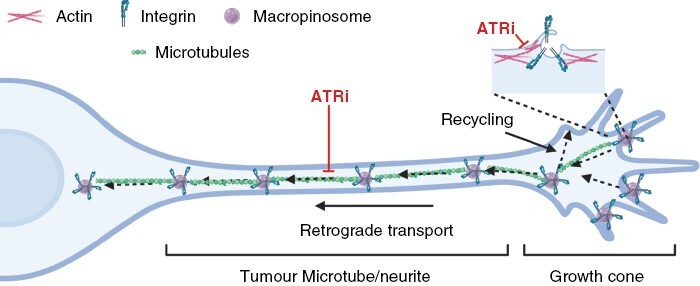

**Key Points**

- Ataxia telangiectasia and Rad 3 related kinase (ATR) expression is elevated in tumor margin regions of human glioblastoma samples and is associated with poorer survival.
- ATR promotes glioblastoma cell invasion via the internalization and trafficking of integrins.
- Pharmacological inhibition of ATR reduces glioblastoma invasion.

**Importance of the study**

Invasion of vital neural structures is the cause of distressing morbidity and a contributing cause of death in glioblastoma patients. Despite infiltration being an obvious therapeutic target, there are no anti-invasive agents currently in clinical use. Ataxia telangiectasia and Rad 3 related kinase (ATR) is a key DNA damage-response protein involved in DNA replication stress and is the focus of intense interest as a potential radiosensitizer in other tumor types. We demonstrate a novel role for ATR in driving glioblastoma cell invasion and propose that ATR inhibitors will have beneficial therapeutic effects in this disease beyond radiation-induced DNA damage potentiation.

Glioblastoma is a treatment-refractory brain tumor with a profoundly invasive phenotype that prevents curative tumor resection. Brainstem invasion by glioblastoma cells is a common feature at autopsy, suggesting that infiltration not only contributes to neurological dysfunction, but also impacts clinical outcome.^1^ Median survival of patients undergoing maximal neurosurgical resection followed by adjuvant radiotherapy (RT) and chemotherapy is only 12–18 months.^2^ Attempts to eradicate invasive microscopic disease by increasing the irradiated volume of the brain have failed to improve survival and caused unacceptable toxicity.^3,4^ A more detailed understanding of the mechanisms involved in glioblastoma invasion has the potential to reveal novel anti-invasive therapies that may enhance the efficacy of existing treatment regimens. Indeed several preclinical studies have indicated the value of this approach, but so far none has successfully translated into the clinic.^5–7^

Ataxia telangiectasia and Rad 3 related (ATR) is a phosphatidylinositol 3-kinase (PI3K)-like kinase which is central to the DNA damage and replicative stress response in the S phase of the cell cycle where it recruits DNA repair machinery and induces S phase arrest.^8–11^ These processes require regulation of nucleoskeletal components to maintain architecture during lesion resolution and to communicate induction of cell-cycle arrest to the cytoskeleton.^12,13^

A noncanonical, cytoplasmic role for ATR has also emerged in recent years. Cara et al. showed that the SCD protein domain targeted by ATR is commonly found in proteins involved in the nervous system, including proteins that regulate vesicle trafficking and cytoskeleton dynamics.^14^ Additionally, several studies implicate ATR in cytoplasmic processes including the maintenance of nuclear plasticity,^15^ and facilitation of endocytosis and excitatory vesicle recycling at neuronal synapses.^16^ The latter observation is of particular interest in glioblastoma cells which share a neural crest lineage and morphological features with neuronal cells, including structures that resemble axonal growth cones at the ends of neurite-like projections called tumor microtubes (TMs).^17^ TMs allow interconnectivity between glioblastoma cells and surrounding neuronal cells, forming a functional network that promotes treatment resistance and tumor cell infiltration.^18,19^

Macropinocytosis is an endocytic process facilitating the cellular uptake of high-molecular-weight extracellular material and is involved in nutrient uptake, antigen presentation, and chemotactic responses.^20,21^ Effective macropinocytosis and trafficking of resultant endosomes require coordinated activity of the actin and microtubule components of the cytoskeleton.^22^ Intriguingly, macropinocytosis at neuronal axonal growth cones determines repulsive axonal turning and retraction.^23,24^ Furthermore, macropinocytosis is implicated in the rapid trafficking of integrins from focal adhesions to cellular ventral surfaces during growth-factor-stimulated cell motility^25^ and in facilitating membrane recycling in neural crest cells to drive migration.^26^ Dysregulated macropinocytosis has been documented in glioblastoma, but to date, a potential link between macropinocytosis and glioblastoma invasion is yet to be investigated.^27,28^

Using in vitro and in vivo studies and 4 different primary glioblastoma lines, we report a novel role for ATR in driving the invasion of glioblastoma cells via the regulation of actin and microtubule networks that support turnover of integrins at growth-cone-like structures. We demonstrate that this function of ATR is highly sensitive to pharmacological inhibition, resulting in abrogation of glioblastoma invasion both in vitro and in vivo, and presents a novel clinical approach to disease management.

## Methods

### Derivation and Maintenance of Primary Glioblastoma Cell Lines

Primary glioblastoma cell lines E2, G7, and R15 were derived from resected tumors (see Supplementary Methods) and maintained as neurospheres as described previously in stem-cell-enriched culture conditions.^29,30^ Cell lines were discarded after 10 passages and were tested for the presence of mycoplasma every 3 months and subjected to STR (Short Tandem Repeat) profiling to confirm authenticity. To provide adherent cells for experiments, stem-cell-enriched neurosphere cultures of G7, E2, R15, and Ox5 were plated on Matrigel-coated plates (0.23 mg/L in AdDMEM, Life Technologies) in stem-cell-enriching media. S24-GFP cells were cultured as neurospheres as previously described.^18^

In vitro irradiation was performed using an Xstrahl RX225 radiation cabinet (195 kV X-rays, dose rate 1.39 Gy/min).

### Integrin Internalization Assays

The assays used to measure integrin internalization have previously been described.^31^ Briefly, cells were labeled with NHS-SS-biotin, and internalization was allowed to continue at 37°C. Cells were then washed and lysed, and levels of biotinylated integrin were determined by capture-ELISA.

### Cell Viability

Cell viability was carried out using CellTiter-Glo according to the manufacturer’s protocol (Promega). Briefly, cells were plated in Matrigel-coated 96-well plates and incubated with incremental concentrations of drugs. Cells were irradiated with 2Gy 1 h after the addition of the drug and incubated for a further 23 h prior to the detection of luminescence (Promega GLOMAX).

### Spatial Transcriptomics

We obtained 16 de novo glioblastoma samples from the Freiburg Spatial Transcriptomics database. The samples were prepared following the protocol outlined in the original publication.^22^ We visualized gene expression levels using the SPATA2 package) in R software. The SPATA2: plotSurface function was used to create 2D plots of gene expression across the tissue section. The color_by and alpha_by parameters were set to the gene expression levels. The display_image parameter was set to TRUE, which generated a visualization of the tissue section overlaid with the gene expression plot. We also applied the smooth function with the standard span factor to generate a smoother representation of the gene expression data. We performed correlation analysis using the SPATAWrappers package.^22^ The runSpatialRegression function was applied with a spatial lag model to identify pairwise correlations between gene expression levels and the abundance of gene signatures across the tissue section. The results of the correlation analysis were visualized using the corplot function, which generated a heat map of the pairwise correlation coefficients.

### siRNA and Plasmid Transfection

Subconfluent G7 cells were transfected with 30 pmol siRNA targeting ATR or control (Dharmacon: cat no J-003202-19-002 Lot no: 190724) using RNAiMAX reagent (Invitrogen 13778-030) and plated into 6-well plate and then incubated for 48 h prior to imaging or protein extractions. For ATR overexpression, G7 cells were transfected with either pcDNA3 or pcDNA3.ATR using SuperFect transcription reagent (Qiagen).

### Subconfluent Migration Assays

Subconfluent migration assays were performed as previously described.^5^

### Immunofluorescence

For confocal imaging, subconfluent glioblastoma cells were plated on coverslips coated with Matrigel and incubated at 37°C for 24 h. For high-throughput imaging, 1 × 10^4^ cells were plated on 96-well black-sided plates (Perkin Elmer) precoated with Matrigel. Cells were incubated with anti-ATR (23HCLC ThermoFisher) or anti-integrin a6 (CD49F 55734 BD Bioscience) antibodies overnight at 4°C followed by incubation with secondary conjugated antibodies, DAPI and HCS Cell Mask Deep Red Stain (ThermoFisher H32721) or Texas red-X Phalloidin (ThermoFisher T7471).

### Confocal and High-Throughput Imaging

Images were acquired using either a Zeiss LSM 780 or 880 confocal microscope and analyzed using Zen 2012 (Zeiss) or Imaris. For high-throughput analysis, images were captured using the Opera Phoenix High-content screening system and analyzed using Columbus Image Analysis software (Perkin Elmer).

### Super-Resolution Microscopy

For macropinocytosis capture, 0.5 × 10^5^ G7 cells were plated on glass-bottomed 35-mm dishes precoated with 5µg/mL fibronectin (ThermoFisher) and allowed to establish for 24 h. Using SuperFect transcription reagent (Qiagen), 1 µg of plasmid containing α5-GFP was then transiently transfected into cells. After 24 h, 0.1 mg/mL lysine fixable, 70 kDa Texas red dextran was added, and cells were allowed to internalize for 30 min before the addition of either DMSO or VE822. For tracking macropinocytosis, time-lapse, super-resolution imaging was performed using Zeiss 880 Airyscan (SR mode) with a Plan-Apochromat 40x/1.3 Oil objective, and images were analyzed using Imaris software. Cells were maintained at 37°C and 5% CO_2_ in an incubation chamber for the duration of imaging.

For EB1 tracking, G7 cells were transfected with pEGFP-EB1 (kindly provided by Prof. Laura Machesky, Cambridge) followed by treatment with DMSO, 1 μM VE822, or 1 μM Bayer 1895344 for 16 h before imaging. Images of EB1-GFP were acquired on a Zeiss Elyra 7 microscope using a 40x NA1.2 water immersion objective and full environmental control to deliver 37°C and 5% CO_2_. Five-phase apotome with z-leap mode was used (0.373-µm spacing). Images were captured onto a pcoEDGE 4.2M sCMOS camera using a 50-ms exposure time. Frame interval between captures was 2 s. Raw images were SIM processed using the “standard” setting in Zen black software.

### Animal Experiments

Animal experiments were performed under the relevant home office license and in accordance with ARRIVE guidelines. All experiments had ethical approval from the University of Glasgow under the Animal (Scientific Procedures) Act 1986 and the EU directive 2010. Mice were maintained in individually ventilated cages with environmental enrichment.

### Intracranial Window Experiments

Male NMRI-*Foxn1*^*nu*^ mice (*n* = 5) were used for this experiment using a previously described protocol.^32^ Two to three weeks after window implantation, mice were injected with 30,000 S24-GFP cells, and tumors were allowed to develop for a further 2–3 weeks.

Mice were given either vehicle (10% vitamin E) or VX970 (50 mg/kg) by oral gavage according to the schedules described in the results section. 200 µL of 10 mg/mL 10 kDa dextran was administered subcutaneously. 10 kDa dextran was used to allow multiple subcutaneous injections and efficient delivery across the blood–brain barrier. 10 kDa dextran can be internalized by other endocytic processes; however, a significant amount is internalized via macropinocytosis.^33^ In vitro uptake assays confirmed that its endocytosis was similarly impacted by ATR inhibition (data not shown). For imaging details, see Supplementary Materials.

### Multiphoton *Intravital* Imaging

Imaging was undertaken using a Zeiss LSM880 NLO multiphoton microscope using W Plan-Apochromat 20x/1.0 NA water immersion objective and subsequently analyzed using Imaris software. Z-stacks were taken to a depth of 60–100 µm, at 1-µm intervals. GFP and TR-dex were excited using a Coherent Discovery “2-photon” laser tuned to 890 nm, CB-dex tuned to 800 nm. Signals were acquired onto GaAsP non-descanned detectors.

### Intracranial Tumor Experiments

Female CD1 nude mice were orthotopically injected with 1 × 10^5^ G7 or S24-GFP cells as previously described^5^ before dosing with either vehicle (10% vitamin E) or 60 mg/kg VX970 and mice culled at the indicated time points. For PK analysis, tumors were sub-dissected and fresh-frozen specimens of blood, tumor, and contralateral hemispheres sent for PK analysis (Vertex). For the invasion study, tumors were allowed to establish for 2-week (S24) or 10-week (G7) mice before daily dosing for 10 days with either vehicle (10% vitamin E) or 50 mg/kg VX970. Formalin-fixed, paraffin-embedded sections were stained for Ki67, and scanned using a Hamamatsu Nanozoomer Slide scanning machine and automated analysis was conducted using QuPath software.

## Results

### ATR Is Found in the Cytoplasm of Glioblastoma Cells, and its Expression Correlates With Invasive Potential

**Figure 1.**
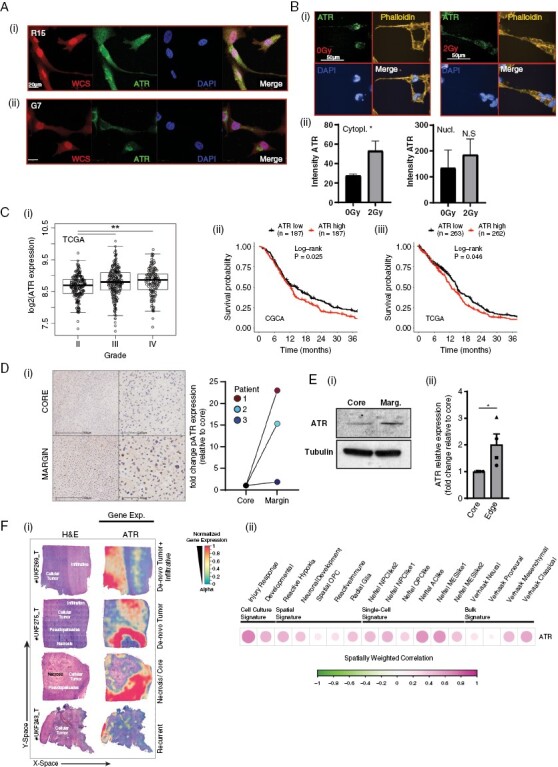
(A) ATR is found in both the nucleus and cytoplasm of glioblastoma cells and is associated with invasive potential. (i) R15 and G7 cells were fixed and stained with whole cell stain , ATR , and DAPI. (B) G7 cells were irradiated with 2Gy and fixed after 6 h and stained for ATR (green), Phalloidin (yellow), and DAPI (blue) followed by high-throughput imaging and automated analysis. Data from 3 biological repeats, 2000–4000 cells imaged per condition per repeat. Bars are mean ± SEM. Statistical analysis using student’s *t*-test. *P < .05, NS = not significant. (C) ATR expression correlates with both (i) glioma grade and (ii), (iii) poorer patient survival. (D) Increased pATR expression can be found in the invasive tumor margin versus the tumor core in patient samples. (i) Core and margin samples were taken from 3 different patients, stained for pATR and scored by a neuropathologist for positive cells and the fold change between core and margin plotted. (E)(i) ATR expression is increased in primary Ox5 glioblastoma cells derived from the tumor margin compared to matched cells from the core. (ii) Western blot quantified from 4 independent biological repeats. Statistical analysis using student’s *t*-test. *P < .05. (F)(i) Spatial transcriptomics identifies increased ATR expression in tumor margins and pseudopallisades (ii) ATR expression was aligned with known cell culture, spatial, single cell, and bulk signatures.

Nuclear ATR activation in response to replication stress has previously been described as a hallmark of glioblastoma cells.^9^ Consistent with previous reports in other cell types, confocal immunofluorescence imaging of glioblastoma cells also showed ATR to also be present in the cytoplasm of primary glioblastoma cell lines (Figure 1A(i, ii)). Cytoplasmic localization was confirmed with a second antibody (Figure S1A), and via subcellular fractionation of lysates from 3 primary glioblastoma lines (Figure S1B). Furthermore, high-throughput imaging and automated quantification of cells subjected to a sublethal dose of radiation (RT; 2Gy), which is known to induce motility in glioblastoma cells,^5–7^ revealed a significant increase in cytoplasmic ATR (Figures 1B(i, ii) and S1B).

Interrogation of 2 publicly available datasets of mRNA expression in glioma (TCGA and CGCA) showed that ATR expression increased with tumor grade, being elevated in highly infiltrative Grade IV tumors compared with lower-grade gliomas (Figures 1C(i) and S1C). Higher ATR expression also correlated with poorer survival in 3 separate datasets (Figures 1C(ii, iii) and S1D). Although the reasons behind these observations are likely manifold, the contribution of infiltration to patient survival^1^ indicates an intriguing link between ATR and tumor invasion.

In 3 human glioblastoma specimens, immunohistochemical studies identified increased pATR expression at the invading edge in matched tumor core and margin samples (Figure 1D(i)). This observation was independently confirmed by western blot analysis of freshly cultured Ox5 primary glioblastoma cells isolated from core and margin tumor regions (Figure 1E(i, ii)). Lastly, we examined the spatial expression profile of ATR utilizing the Freiburg spatial transcriptomic atlas, which comprises 16 de novo glioblastoma specimens.^34^ ATR expression was observed to be associated with histological regions, including pseudopalisading, perinecrotic areas, and microvascular proliferation, as classified by the Ivy glioblastoma atlas. These characteristic glioblastoma structures are linked to increased invasive capacity (Figure 1F(i)). To further investigate the spatial correlation between ATR gene expression and the abundance of known expression subgroups, we applied a spatial lag model for correlation analysis. ATR expression demonstrated variability across subtypes but exhibited increased levels in subtypes associated with enhanced migration/infiltration, such as injury response and Neftel MES 1 (Figure 1F(ii)).^35^

These results, from 3 independent datasets, indicate a potentially clinically relevant association between ATR expression and infiltration.

### ATR Is Required to Drive Glioblastoma Cell Migration In Vitro

**Figure 2.**
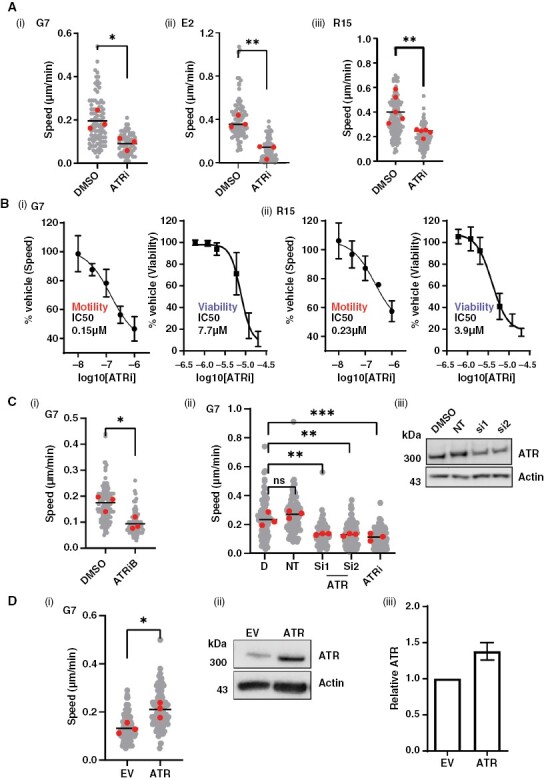
ATR is required to drive glioblastoma cell migration in vitro. A(i) G7, E2, and R15 cells were plated subconfluently and treated with either DMSO or ATRi (1 µM VE822) for 24 h followed by time-lapse microscopy and single-cell tracking. (B) G7 (i) and R15 (ii) cells show a dose-dependent reduction in migration speed with ATR inhibition, at sublethal concentrations of inhibitor. Cells were incubated with DMSO/ATRi for 24 h followed time-lapse microscopy and single-cell tracking or viability assay. (C)(i) G7 cells were plated subconfluently and treated with either DMSO or ATRiB (1 µM Bayer 1895344) for 24 h followed by time-lapse microscopy and single-cell tracking. (ii) G7 cells were treated with siRNAs targeting ATR or 1 µM ATRi (VE822) followed by time-lapse microscopy and single-cell tracking. ATR knockdown was confirmed by western blot (iii). D = DMSO; NT = non-targeting siRNA. (D)(i) ATR was overexpressed in G7 cells followed by time-lapse microscopy and single-cell tracking. (ii) and (iii) Overexpression was confirmed by western blot (*n* = 2, error bar = std dev). (ii). EV = Empty vector (pcDNA3). For all motility assays, data from 3 biological repeats, >30 cells tracked per repeat (gray). Two-tailed, unpaired Student’s *t*-test was performed on means from each biological repeat . For siRNA in C(ii), a 1-way ANOVA and Dunnett’s test were performed. *P < .05, **P < .01, ***P < .001, n.s. = nonsignificant.

To investigate the functional role of cytoplasmic ATR, 3 primary glioblastoma lines (G7, E2, and R15) were treated with the ATR inhibitor (VE822/VX970/Berzosertib), referred to as ATRi henceforth, followed by subconfluent migration assays (Figure 2A(i–iii)). ATRi significantly reduced migration speed of all three glioblastoma lines at concentrations >10-fold below those required to impact cell viability after 24-h exposure (Figures 2B(i, ii) and S2A; Table S1). These data suggest that ATR has a specific role in regulating the invasive potential of glioblastoma cells that is independent of its role in DNA damage response. As RT is given as a standard of care to all glioblastoma patients, and radiation has previously been reported to induce invasion of glioblastoma cells,^5–7^ we repeated the experiment following treatment of cells with 2Gy radiation. Interestingly, we observed an increased sensitization of 2 out of 3 of the cell lines after radiation (Table S1).

VE822/VX970 is a well-characterized and highly specific inhibitor of ATR, and its efficacy as an anti-invasive compound at low concentrations suggests that our observations reflect on-target effects of the inhibitor. However, to confirm this, we performed subconfluent migration assays following exposure to both an additional ATR inhibitor, Bay1895344 (ATRiB), in all 3 lines (Figures 2C(i) and S2B), and siRNAs and CRISPR deletions targeting ATR in the most genetically tractable glioblastoma line, G7 (Figures 2C(i, ii) and S2C). The results demonstrated a significant decrease in migration speed under all conditions, providing confirmation of an on-target effect of ATR inhibition on motility. Western blots for pChk1 (a nuclear target of ATR) were conducted to ensure drug efficacy at the chosen concentration (Figure S2D). The motility data were further confirmed using a more complex 3D invasion assay using Matrigel-coated scaffolds plated with E2 and R15 cells (Figure S2E(i, ii)).^36^

The above data strongly indicate that ATR is required to support glioblastoma cell migration in vitro. To ascertain whether ATR is also able to actively drive migration, we overexpressed ATR in G7 cells. We observed a significant increase in migration speed, indicating that ATR may indeed play an active role in migration induction (Figure 2D(i–iii)). Interestingly, preliminary data suggest that the role in invasion may be unique to ATR, since inhibiting 2 other DNA damage-response factors (PARP and ATM) had no effect on migration in any of the 3 cell lines tested (Figure S2F).

### Inhibition of ATR Increases Cytoplasmic Macropinosomes

**Figure 3.**
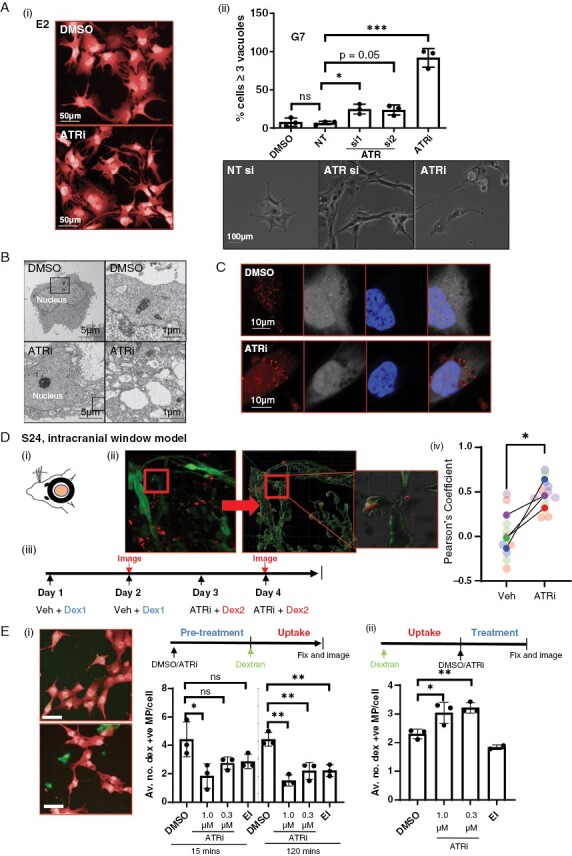
Inhibition of ATR causes a decrease in active macropinocytosis and endocytic processing. (A)(i) An accumulation of large vacuoles following either treatment with ATRi was observed in all 3 cell lines, E2 shown as an example. (ii) Number of vacuoles per cell following ATRi or RNAi of ATR was quantified in G7 cells, statistical significance was determined using a 1-way ANOVA and Dunnett’s test. (B) Electron microscopy of G7 cells showed vacuoles to be single membrane bound. (C)(i) E2 cells incubated with 70 kDa Texas red dextran (red) alongside ATRi or DMSO. White = whole cell stain, blue = DAPI. (D) Treatment of mice with ATRi (VX970) in an intracranial window model causes an accumulation of labeled dextran in vivo. (i) Multiphoton imaging and processing were used to detect the uptake of fluorescent dextran in to GFP-S24 cells in the mouse brain. (ii) Five mice were treated with DMSO and Cascade blue labeled dextran followed by imaging, then treated with ATRi and Texas red labeled dextran followed by second imaging in a longitudinal experiment (1 mouse received only ATRi plus CB-Dex). (iii) Pearson’s coefficient was calculated under each treatment condition for each mouse to estimate colocalization of dextran with S24-GFP cells using Imaris colocalization tool. >3 images per imaging session per mouse, image data plotted individually (small points) or as mean for each mouse (large points), color coded for each mouse. **P < .01, Student’s *t*-test. (E) Timed experiments demonstrate ATRi inhibits both the uptake and downstream degradation of internalized dextran. (i) G7 cells were incubated with DMSO, ATRi, or EIPA for 15 min or 2 h before incubation with labeled dextran. (ii) G7 cells were pre-incubated with dextran to allow uptake before addition of DMSO, ATRi, or EIPA. Data represents 3 biological repeats, 2000–4000 cells quantified via high-throughput imaging per condition per repeat. Statistical significance was determined using a 1-way ANOVA and Dunnett’s test. *P < .05; **P < 0.01, n.s = not significant.

In addition to migration defects, treatment with ATRi-, ATRiB-, or siRNA-mediated knockdown of ATR increased the appearance of enlarged vacuolar structures within the cytoplasm of glioblastoma cells (Figures 3A(i, ii) and S3A). We conducted a series of experiments to elucidate the origin of these structures. Autophagy has been demonstrated to modulate cell migration and integrin membrane recycling.^37^ To investigate whether dysregulation of autophagy was underpinning our observations, we undertook autophagic flux assays as previously described.^38^ Western blot analysis for processed LC3B from cells treated with/without VE822 and chloroquine indicated no change in the levels of LC3B II, indicating that the observed vacuoles were unlikely to be derived from a block in autophagy (Figure S3B). Indeed, electron microscopy revealed the vacuoles to be single membrane bound, inconsistent with autophagosomes (Figure 3B). These data demonstrate that autophagy likely does not contribute to the observed phenotype.

Next, we investigated whether these structures could be macropinosomes, which are characteristically large (0.2–5 µm) and bound by a single membrane. G7 cells were incubated with a Texas red labeled 70 kDa dextran (red) in the presence of ATRi. Dextrans of this size are predominantly internalized via macropinocytosis, and are an established method to mark cellular structures that result from this endocytic process.^39^ The cells were fixed, stained, and subjected to confocal imaging (Figure 3C(i, ii)). Indeed, a large proportion of the vacuoles contained dextran, identifying them as macropinosomal in origin. Localization was confirmed as intracellular via z-stack reconstruction (Figure S3C).

To confirm biological relevance, we used orthotopic *intravital* imaging to look for dextran accumulation in GFP-S24 cells in vivo (Figure 3D(i)). Using advanced imaging and processing with Imaris software, we were able to confidently detect internalized fluorescent dextran within GFP-S24 cells within the mouse brain (Figure 3D(ii)). The longitudinal nature of this technique allows each mouse to be used as its own control. Mice (*n* = 5) were initially treated on 2 consecutive days with a vehicle alongside cascade blue-dextran (CB-dex), before multiphoton imaging. They were then treated twice with ATRi alongside Texas red dextran (TR-dex), before repeat imaging (Figure 3C(iii)). By changing the color of the dextran label, we ensured that only dextran internalization/processing that may have been affected by ATRi treatment was detected in the second imaging session. To control for any variance in fluorophore detection affecting results, the order of administration of dextran colors was switched in one mouse (TR-dex/veh then CB-dex/ATRi), and an additional mouse was treated with ATRi and CB-dex alone. Subsequent colocalization analysis revealed that ATRi caused an accumulation of dextran in GFP-S24 cells, confirming the biological relevance of our in vitro findings (Figure 3D(iv)).

### ATRi Causes a Block in Both Active Macropinocytosis and Processing of Internalized Macropinosomes

To test whether the increase in macropinosomes was due to an increase in active macropinocytosis or a block in the processing of internalized macropinosomes, we conducted a pair of timed experiments. First, cells were treated with either DMSO, ATRi, or an inhibitor of macropinocytosis (EIPA, positive control) for 15 min or 2 h prior to the addition of 70 kDa Fluorescein-dex. Cells were allowed to internalize dextran for 30 min before fixing (at 15 or 120 min), staining, and high-throughput imaging and automated analysis (Figure 3E(i)). We observed a significant dose-dependent decrease in dextran-positive macropinosomes in ATRi and ATRiB pretreated cells using all 3 cell lines (Figures 3E(ii) and S3D(i, ii)), indicating a block in active macropinocytosis.

With inhibition of active macropinocytosis being observed upon pretreatment with ATRi (Figure 3E(ii)), but a clear accumulation of dextran-positive macropinosomes apparent when ATRi and dextran are given concomitantly (Figure 3C), we concluded that a block in the lysosomal processing of cytoplasmic macropinosomes, and thus degradation of dextran, must also be occurring. To confirm this, cells were allowed to internalize dextran for 30 min in the absence of compound, prior to its removal and addition of DMSO, ATRi, or EIPA for 15 min (Figure 3A(ii)). Analysis showed a turnover of pre-internalized dextran is reduced in a dose-dependent manner in cells treated with ATRi, strongly suggesting that a block in processing is the cause of intracellular dextran accumulation.

Together, these data indicate that blocks in active macropinocytosis and macropinosome processing occur in tandem after ATR inhibition. It is important to note that, while using 70 kDa dextran allows us to follow macropinocytosis, ATRi may also be impacting on other types of endocytosis.

### Macropinocytosis Is Required to Internalize Integrins at TM Growth-Cone-Like Structures, Allowing TM Deadhesion

**Figure 4.**
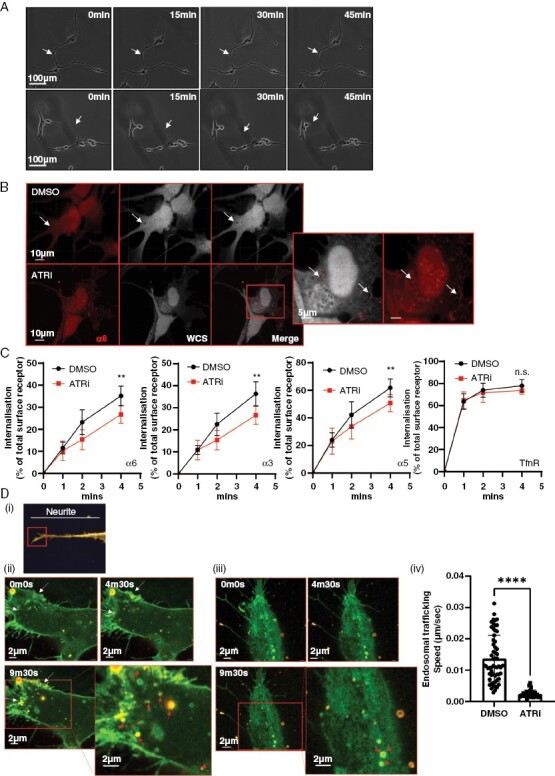
Macropinocytosis is required to internalize integrins at TM growth-cone-like structures, allowing TM deadhesion, retraction, and cell migration. (A) Time lapse of migrating G7 cells following 24 h of ATRi/DMSO exposure. Arrows indicate the termini of neurites. (B) E2 cells were treated with DMSO or 1 µM ATRi for 5 h, followed by fixing and staining for WCS (white), Integrin α6 (red). (C) G7 cells were treated with DMSO or 1 µM ATRi before lysis and measurement of internalized pool of integrins. Data from 3 independent experiments. **P > 0.01, Mann–Whitney. (D)(i) Super-resolution microscopy was used to image the growth-cone-like structures at the ends of glioblastoma neurites. (ii) G7 cells expressing GFP-α5 integrin were incubated with 70 kDa Texas red dextran followed by DMSO (ii) or VE822 (iii) prior to super-resolution, time-lapse imaging of growth cones. See Fig S4C for images of separated fluorescence channels. Yellow = colocalization. White arrows indicate areas of membrane retraction; red arrows indicate dextran-positive macropinosomes. (iv) Speed of intracellular trafficking of endosomes was measured using automated tracking in Imaris. Data from 3 super-resolution images of growth cones per condition. Statistical analysis Student’s *t*-test ****P > .001

Time-lapse imaging of treated glioblastoma cells revealed a deadhesion/retraction defect at the termini of neurite structures upon ATR inhibition, resulting in their increasing lengthening and fragility over time, leading to destabilization and ultimately breakage. (Figures 4A and S4A; SV 1, 2). This supports the observation in Figure 3D, where TM length was shown to be significantly shorter after 24 h of ATRi treatment.

The parallel observations of a deadhesion/retraction defect caused by an inability of TMs to release from the extracellular matrix (ECM), plus a block in macropinocytosis are intriguing and raise the possibility of a mechanistic connection between the 2 phenomena.

Immunofluorescent staining of E2 cells treated with ATRi revealed enrichment of integrin α6 on the surface of the stalled macropinosomes (Figure 4B). We hypothesized that macropinocytosis may be required for the rapid release of neurite growth-cone-like structures through integrin internalization and hence allow active migration of glioblastoma cells. This hypothesis is supported by observed rapid adhesion/deadhesion in the highly dynamic growth-cone-like structure of glioblastoma cells grown in 3D, suggestive of a requirement for bulk trafficking of integrins.

To investigate this hypothesis, we performed internalization assays for α5, α3, and α6 integrins. Transferrin R (TrnR) is passively, as opposed to actively, internalized and was used as a negative control. The data in Figure 4C(i–iv) clearly indicate that internalization of all 3 integrins is reduced upon treatment with ATRi, providing further evidence of the role of ATR in regulating integrin internalization. The reduction is modest, suggesting that internalization may be occurring in a discrete location in the cell that is, at the growth-cone-like structures at the ends of neurite protrusions.

To investigate this, we overexpressed GFP-labeled α5 integrin in G7 cells and used super-resolution, time-lapse microscopy to look at 70 kDa dextran uptake at growth-cone-like structures at the ends of neurites (Figure 4D(i)). Cells were incubated in TR-dex before the addition of DMSO or ATRi. In control cells, we obtained clear evidence that macropinocytosis was occurring at areas of membrane retraction, and that many of the resulting macropinosomes were positive for both GFP-α5 integrin and TR-dex (confirmed by imaging at higher temporal resolution, data not shown; Figure 4D(ii), SV3; separated fluorescence panels in Figure S4B). In addition to a subset of larger macropinosomes that were largely static, we observed a population of highly mobile smaller macropinosomes consistent with observations from studies in neuronal crest cells.^26^ We also observed dextran-negative endosomes of a range of sizes, some of which are likely too small to be macropinocytotic in origin.

Importantly, we observed that treatment with ATRi downregulated de novo macropinocytosis, with all pre-existing, internalized macropinosomes becoming static (Figure 4D(iii, iv); SV4; separated fluorescence panels in Figure S4B). This latter observation indicates defective trafficking of the structures through the cytoplasm, either to be recycled to the membrane or shuttled to the lysosomes for degradation.

These data provide strong evidence to support our hypothesis that ATR is required for the effective internalization of integrins and subsequent deadhesion and retraction of TMs to enable effective migration in vitro and in vivo.

### ATRi Causes a Reduction in Actin Cytoskeleton and Microtubule Plasticity

**Figure 5.**
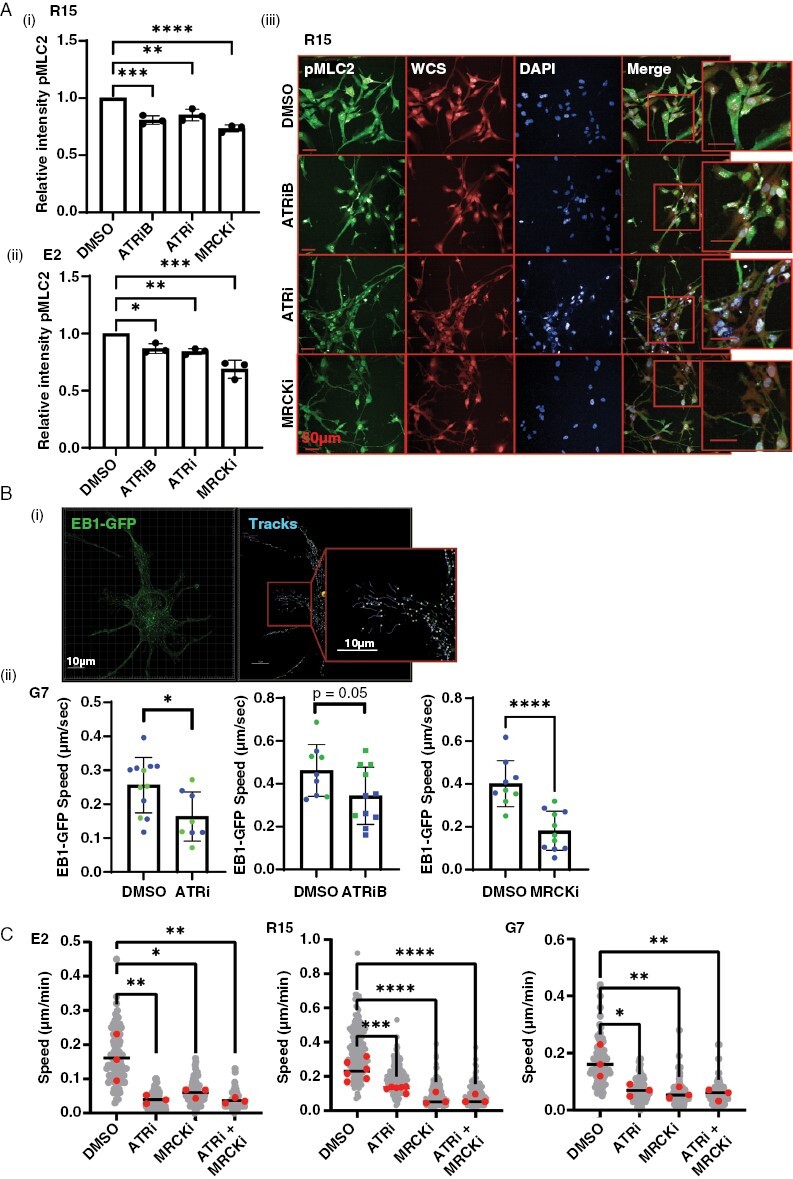
ATRi causes a reduction in actin cytoskeleton and microtubule plasticity. (A) R15 and E2 cells were incubated with DMSO, 1 µM ATRiB, 1 µM ATRi, or 1 µM MRCKi for 6 h before staining with antibody against pMLC2 , whole cell stain , and DAPI followed by high-throughput imaging and automated analysis. Images from R15 shown as an exemplar. Data from 3 biological repeats, 2000–4000 cells imaged per condition each repeat. Bars are mean ± SD. Statistical significance was determined using one way ANOVA and Dunnett’s test: **P > .01, ***P > .005. (B)(i) EB1-GFP was transiently expressed in G7 cells, incubated with DMSO, ATRi, ATRiB, or MRCKi for 16 h (ii), followed super-resolution time-lapse imaging and 3D automated tracking of EB1-GFP foci in Imaris. EB1-GFP foci tracking data from two biological repeats (color coded). Each point represents data from a single image (1–3 cells) with >100 EB1-GFP foci tracked per image. *P < .05, Student’s *t*-test. (C) G7, E2, and R15 cells were plated subconfluently and treated with either DMSO, ATRi (1 µM VE822), MRCKi (1 µM BDP9066), or combined treatment for 24 h followed by time-lapse microscopy and single-cell tracking. Statistical analysis = 1-way ANOVA; *P < .05, **P < .01, ***P < .001, ****P < .0001.

Since our results indicate that ATRi inhibits cell motility, active macropinocytosis, and endosomal processing, we hypothesized that ATRi may be inhibiting actin cytoskeleton dynamics, which support these processes. We examined actin cytoskeleton dynamics by treating E2 and R15 cell lines with DMSO, ATRi, or ATRiB followed by high-throughput imaging after immunofluorescent staining for pMLC2, a marker of actin cytoskeleton contractility. An inhibitor of MRCK (BDP9066) was used as a positive control.^5^ We observed a significant decrease in pMLC2 levels in both cell lines after treatment with both ATR inhibitors, indicating a reduction in actin cytoskeleton contractility (Figure 5A(i–iii). Total MLC2 was unchanged (Figure S5).

As we also observed a reduction in endosomal trafficking, we looked for the involvement of the microtubule network in our observed phenotypes. Microtubules serve as tracts to allow trafficking of intracellular cargo, including endosomes. EB1 binds to the plus end of microtubules and can be used to track their dynamic growth.^40^ GFP-EB1 was transiently expressed in G7 cells followed by treatment with either DMSO, ATRi, ATRiB, or MRCKi for 16 h. Time-lapse confocal imaging was undertaken to capture EB1 dynamics (Figure 5B, SV5). Automated tracking software was used to track XYZ EB1-GFP foci position over time and measure average speed (Figure 5B(ii); SV6). Treatment with both inhibitors significantly reduced EB1 foci speed, indicating that the microtubule network is also impacted by ATRi. Although MRCK is primarily an inhibitor of actin–myosin dynamics and does not act directly on the microtubule networks, the substantial crosstalk between the two cytoskeletal components causes microtubule dysregulation following prolonged MRCKi (16 h), and therefore, we used this inhibitor as a positive control. We also treated cells with the microtubule polymerization inhibitor, nocodazole, and saw rapid loss of EB1 foci, confirming the specificity of our tracking experiments (data not shown).

These data strongly indicate that ATRi inhibits cell motility, macropinocytosis, and endosomal trafficking by reducing the dynamics of the actin cytoskeleton and microtubule networks. As further confirmation, we performed subconfluent migration assays following treatment with DMSO, ATRi, MRCKi, or combination treatment with both inhibitors (Figure 5C). We saw no evidence of an additive or synergistic effect, suggesting that ATRi does indeed abrogate migration via downregulation of cytoskeletal activity.

### Inhibition of ATR Opposes Glioblastoma Cell Infiltration In Vivo

**Figure 6:**
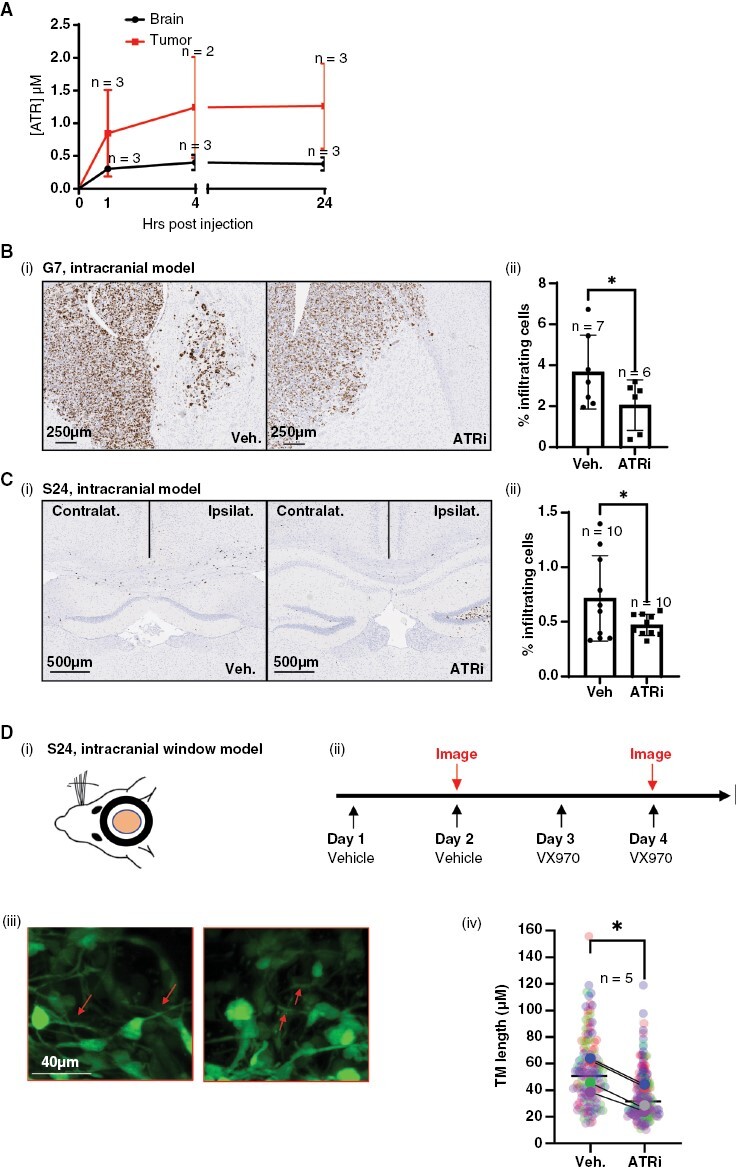
Inhibition of ATR opposes glioblastoma cell infiltration in vivo. (A) Pharmacokinetic (PK) analysis of VX970 delivery to G7 intracranial tumors and normal brain (contralateral hemisphere). (B)(i) Intracranial injection of G7 cells was undertaken and tumors allowed to develop for 10 weeks, followed by 2 weeks of treatment with vehicle or VX970. Percentage Ki67-positive glioblastoma cells outside tumor bulk were calculated using automated analysis. (C)(i) Intracranial injection of S24 cells and tumors allowed to develop for 2 weeks followed by 2 weeks of treatment with vehicle or VX970 and cull of animals at a timed endpoint and processed as in (B). For both B and C, 2–4 sections per mouse were analyzed and averaged, and each average/mouse plotted as a single data point. *P < .05. (D)(i) Intracranial window mice with S24 tumors were dosed and imaged according to the schedule in (ii). Tumor microtube (TM) length was measured in multiple images per mice and image data plotted individually (small points) or as mean for each mouse (large points), color coded for each mouse. Arrows indicate TM structures. *N* = 5, 1 mouse only received VX970, **P > .001. For all experiments, the number of mice per cohort is indicated adjacent to the relevant data point.

Initial pharmacokinetic studies demonstrated tumor penetration of VX970 in an intracranial model of glioblastoma (G7) at levels sufficient to inhibit the kinase and induce phenotypic effects (Figure 6A). Importantly, drug delivery was enhanced in the tumor compared to the contralateral brain, thereby enhancing tumor specificity. We tested the ability of ATRi to inhibit glioblastoma invasion in vivo using the same intracranial model (G7). Tumors were allowed to develop for 10 weeks and then treated with ATRi and sacrificed (Figure 6B(i)). The brains were fixed and sectioned then stained for Ki67 to label proliferating glioblastoma cells within the nonproliferative brain tissue, using a previously validated method (via correlation with human leukocyte antigen for quantifying invasion.^5^ Subsequent automated analysis revealed that invasion was significantly decreased in mice treated with ATRi (Figure 6B(ii)). To validate this result, we repeated the experiment using the highly infiltrative S24 cell line (Figure 6C(i)) and observed a significant decrease in the percentage of invading glioblastoma cells in the contralateral hemisphere following treatment with ATRi (Figure 6C(ii)).

Finally, the intracranial window model was employed to monitor morphology pre- and post-treatment. Mice (*n* = 5) were treated twice with the vehicle, followed by multiphoton imaging of tumor cells within the brain. The same mice were then treated twice with ATRi followed by repeat multiphoton imaging (Figure 6B(ii)). Measurement of TM length under control and treatment conditions revealed a significant decrease in length indicative of reduced invasive potential (Figure 6B(iii, iv)).

## Discussion

We present data that describe a novel, noncanonical role for ATR kinase in facilitating tumor cell motility and invasion via macropinocytic internalization of integrins and identify ATR as a readily translatable target to achieve containment of disease dissemination in glioblastoma.

Inhibiting invasion has been shown to correlate with increased mouse survival in previous studies using different glioblastoma models.^5,6^ We show with 3 separate in vivo experiments that inhibition of ATR with VX970 effectively abrogates invasion. In vitro we demonstrate the efficacy of 2 separate ATR inhibitors, as well RNAi, in inhibiting glioblastoma cell motility. Furthermore, we show that overexpression of ATR can drive increased motility, conferring on ATR an active role in the regulation of cell migration.

Our novel observation linking macropinocytosis to invasion in glioblastoma opens an intriguing and exploitable therapeutic vulnerability. Macropinocytosis is involved in the bulk trafficking of integrins to allow active migration and is a prominent feature in glioblastoma.^27,28,41,42^ The importance of macropinocytosis in growth cone deadhesion and neurite retraction/redirection has previously been described in neuronal cells.^23,24^ TMs of glioblastoma cells share multiple properties with the neurite projection of neuronal cells, including the presence of growth cones, the ability to probe and respond to the local environment through dynamic movement, and even the ability to form functional synapses.^17–19^ Glioblastoma cells protrude long TMs into the normal brain that one could postulate are sensing the new environment, before committing to invasion. In this situation, the ability to deadhere rapidly to allow retraction or redirection in response to positive or negative environmental cues would be advantageous. Indeed, macropinocytosis has also been implicated in chemotaxic response and nutrient sensing.^20,21^

While the canonical functions of ATR in replication stress and DNA damage signaling in glioblastoma are well characterized, we demonstrate for the first time that ATR is required to mediate internalization of integrins and subsequent trafficking of endosomes to allow active migration in glioblastoma cells.^14–16^ Upon first consideration, this observation may be surprising. However, the ability of ATR to trigger cell-cycle arrest and modulate kinesins and cytoskeletal dynamics provides a compelling mechanistic link.^14,43,44^ Indeed, our study presents the novel observation that ATR modulates cytoskeletal dynamics in glioblastoma cells, beyond the previously described context of the cell cycle.

ATR inhibitors are the subject of intense interest due to their potentiation of DNA damage in combination with conventional cytotoxic agents and radiation in a range of tumor sites. In the context of neuro-oncology, the ATR/replication stress response axis is a particularly appealing target. Treatment resistance in glioblastoma is driven by a subpopulation of radioresistant glioma stem cells and ATRi has been shown to be a potent radiosensitizer in vitro.^30^ Our novel findings suggest a dual clinical benefit to ATRi in both reducing tumor burden and containing invasion. It could be envisaged that ATRi could also be employed in an adjuvant setting to reduce malignant infiltration into vital neural structures.

In summary, we present a novel role for ATR in regulating glioblastoma invasion which, in addition to furthering our understanding of the mechanisms underpinning glioblastoma invasion, has significant relevance for clinical translation. We predict that this previously unappreciated function of ATR will lead to unexpected clinical benefits from therapeutic strategies combining ATR inhibition with RT.

## Supplementary Material

noad210_suppl_Supplementary_Data

noad210_suppl_Supplementary_Figure_S1

noad210_suppl_Supplementary_Figure_S2

noad210_suppl_Supplementary_Figure_S3

noad210_suppl_Supplementary_Figure_S4

noad210_suppl_Supplementary_Figure_S5

noad210_suppl_Supplementary_Material

noad210_suppl_Supplementary_Table_S1

noad210_suppl_Supplementary_Video_S1

noad210_suppl_Supplementary_Video_S2

noad210_suppl_Supplementary_Video_S3

noad210_suppl_Supplementary_Video_S4

noad210_suppl_Supplementary_Video_S5

noad210_suppl_Supplementary_Video_S6

## Data Availability

Data will be made available upon reasonable request. Pre-print published on ResearchSquare: https://doi.org/10.21203/rs.3.rs-967109/v2
