# Supplementary material for: Inhibition of ATR opposes glioblastoma invasion through disruption of cytoskeletal networks and integrin internalization via macropinocytosis
Source: Neuro Oncol. 2023 Nov 4;26(4):625–39. doi: 10.1093/neuonc/noad210 (PMC10995506; doi:10.1093/neuonc/noad210)
Supplement: noad210_suppl_Supplementary_Data [file noad210_suppl_supplementary_data.docx]

**Figure S1. A)** G7 cells were fixed and stained with an alternate antibody against ATR (green) and for actin (yellow). **B)** Cell lysates harvested fron G7, E2 and R15 cells were subject to subcellular fractionation and subsequently blotted for ATR, pATR, GAPDH (cytoplasmic positive control) and Histone H3 (nuclear positive control). **C) and D)** Confirmation of the findings in Fig 1C using additional data sets.

**Figure S2: A)** E2 cells were incubated with DMSO/ATRi for 24 hours followed by subconfluent migration assay or viability assay. **B)** E2 and R15 cells were incubated with DMSO/ATRiB (1μM or 3 μM Bay1895344) for 24hrs followed by subconfluent migration assay. Data from 3 biological repeats. Statistical analysis student TTest. **C)** ATR was deleted from G7 cells using a CRISPR-Cas9 system followed by subconfluent migration assay. Data from three biological repeats. **D)** G7 cells were irradiated with 5Gy and incubated with DMSO or increasing concentrations of ATRi (**(i);** VE822) or ATRiB (**(ii);** Bay1895344) before western blot analysis for pChk1 **E) (i)** Scaffold invasion assay. **(ii)** E2 and R15 cells were plated on Matrigel coated scaffolds and allowed to invade for 5 (R15) or 7 (E2) days before fixation, staining , highthroughput imaging and quantification using Columbus software. Data from 3 (DMSO, ATRi, MRCKi) or 2 (ATRiB) biological repeats each containing 6 internal replicates. Statistical analysis: one-way anova. **F)** G7, R15 and E2 cells were incubated with DMSO, 1 μM Olaparib (PARPi) or 1 μM AZD1390 (ATMi) for 24hrs followed by subconfluent migration assay. Data from 3 biological repeats. Statistical analysis one-way anova. For all data *p<0.05, ** p<0.01, *** p<0.001, n.s. = non-significant.

**Figure S3: A)** E2 cells were fixed and stained with whole cell stain after treatment with DMSO or ATRiB. **B)** Western blot analysis for processed LC3B from cells treated with/without VE822 and chloroquine indicated no change in LC3NII levels. **C)** E2 cells incubated with ATRi and 70kDa Texas red dextran before fixation, staining and imaging. 3D reconstruction using Imaris software. **D)** Indicated cell lines were pre-incubated with (i) DMSO, ATRi or EIPA (statistical analysis = one way anova) or (ii) DMSO or ATRiB (statistical analysis = students ttest), before addition of Texas red 70kDa dextran. Data represents 3 biological repeats, 2000-4000 cells quantified via high throughput imaging and analysis per repeat. **p*<0.05, ***p*< 0.01

**Figure S4: A)** Timelapse of migrating G7 cells following 24 hours of ATRi/DMSO exposure. Arrows indicate the termini of neurites. **B)** Separated fluorescence channels for the final timepoint in Fig 4D.

**Figure S5: A)** R15 and E2 cells were incubated with DMSO, 1µM ATRiB, 1µM ATRi or 1µM MRCKi for 6 hours before lysis and western blotting for total MLC2 and actin.
