# Supplementary figures and images for: Inhibition of ATR opposes glioblastoma invasion through disruption of cytoskeletal networks and integrin internalization via macropinocytosis
Source: Neuro Oncol. 2023 Nov 4;26(4):625–39. doi: 10.1093/neuonc/noad210 (PMC10995506; doi:10.1093/neuonc/noad210)

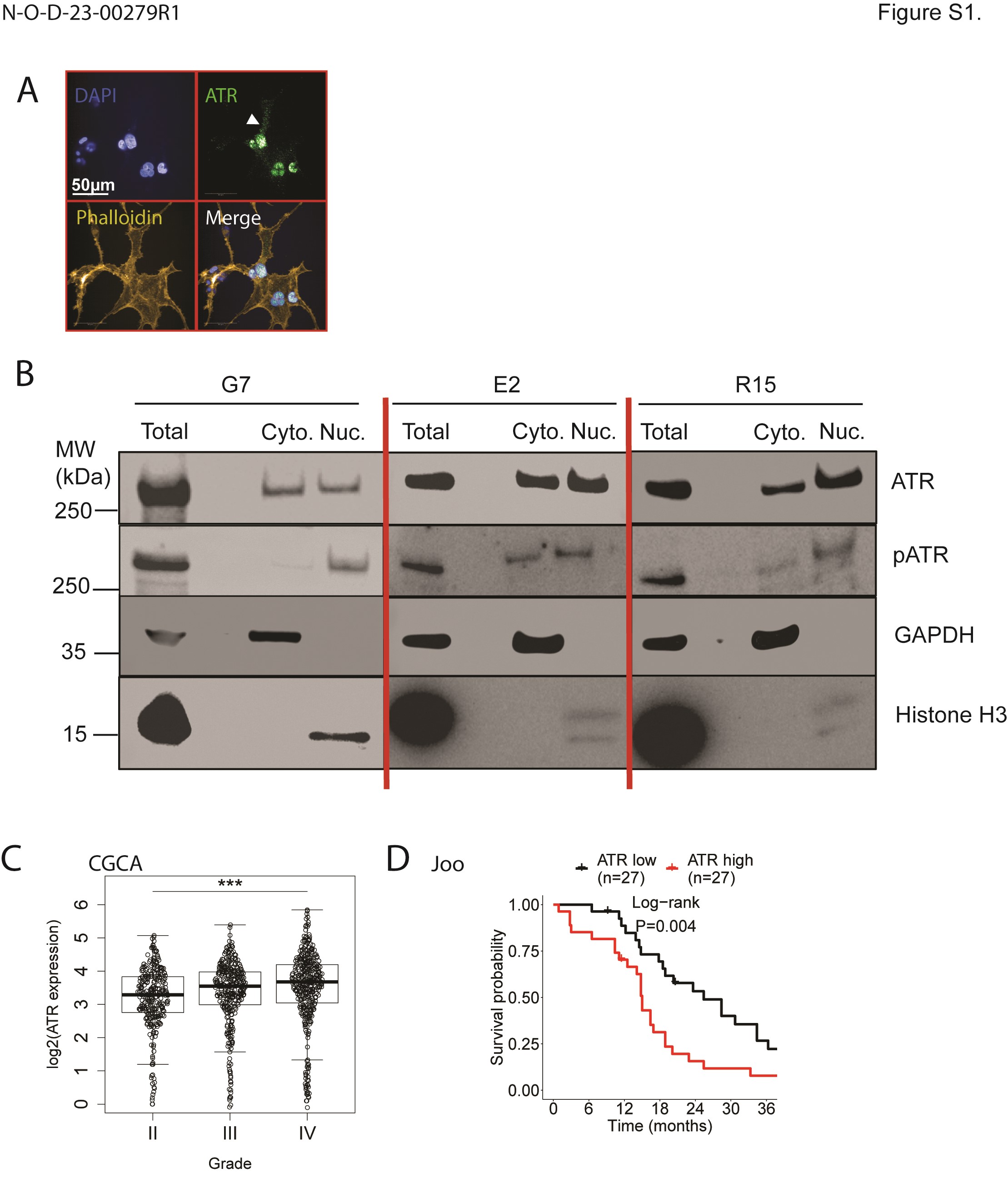

Supplement: noad210_suppl_Supplementary_Figure_S1 [file noad210_suppl_supplementary_figure_s1.jpeg]

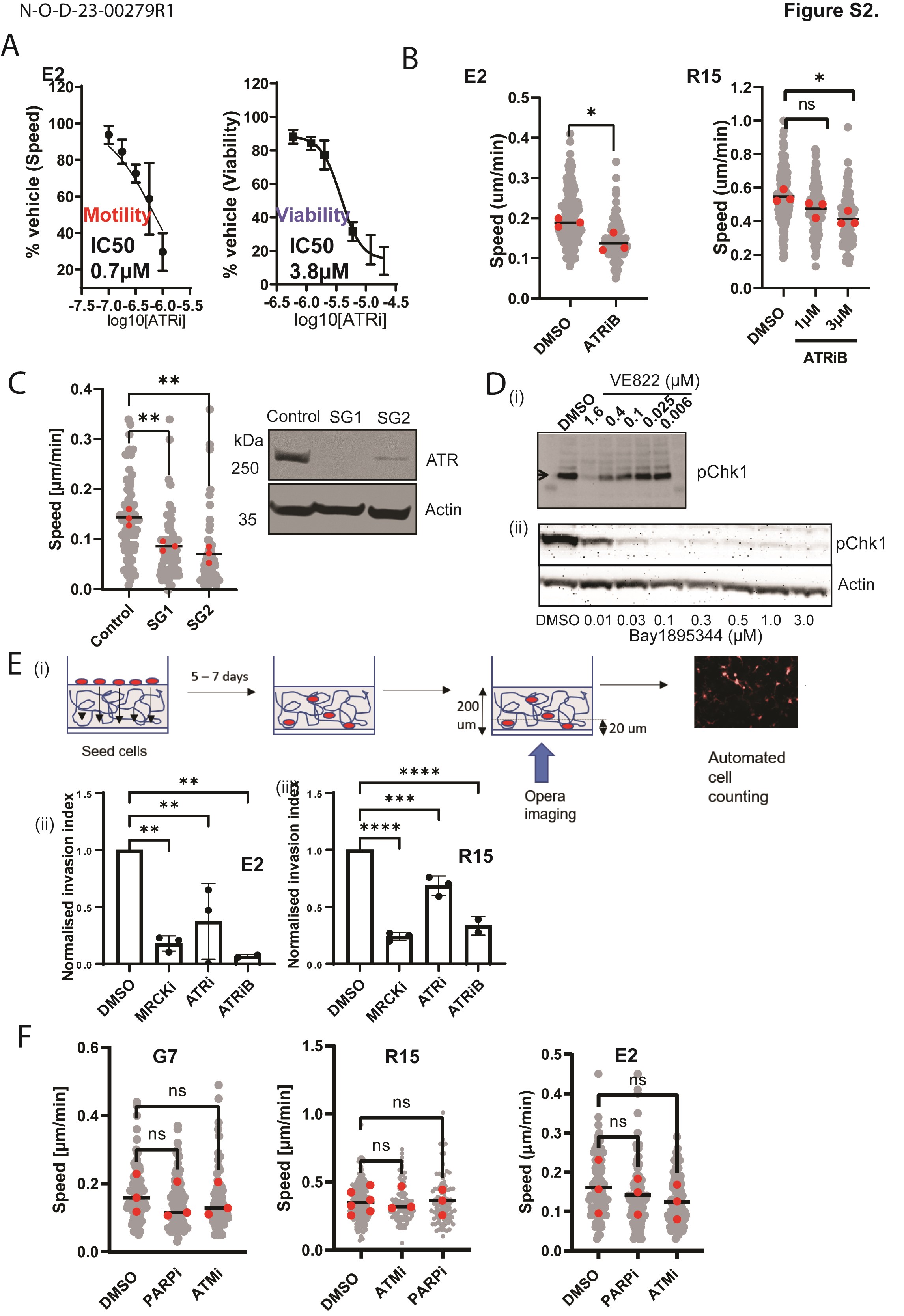

Supplement: noad210_suppl_Supplementary_Figure_S2 [file noad210_suppl_supplementary_figure_s2.jpeg]

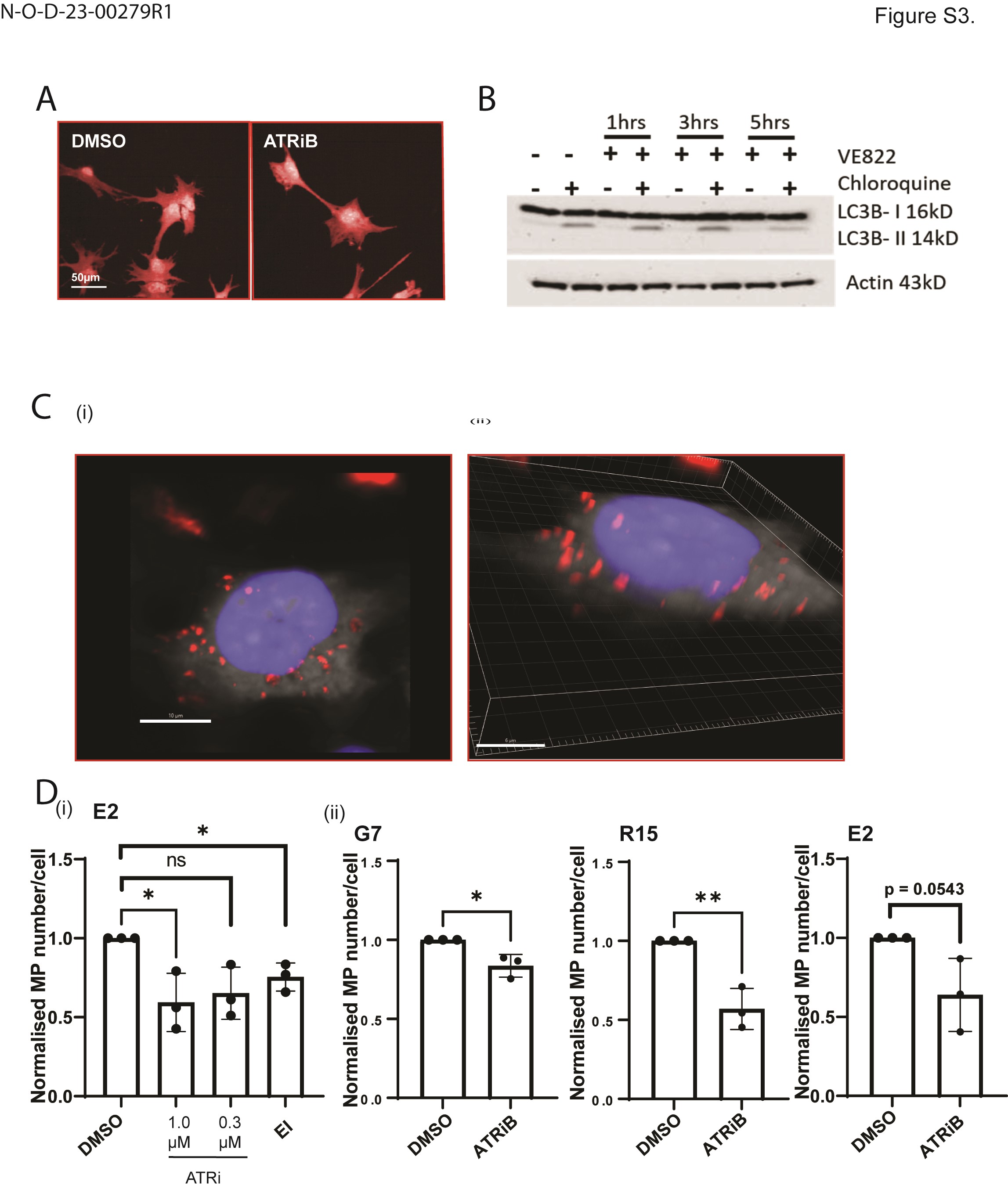

Supplement: noad210_suppl_Supplementary_Figure_S3 [file noad210_suppl_supplementary_figure_s3.jpeg]

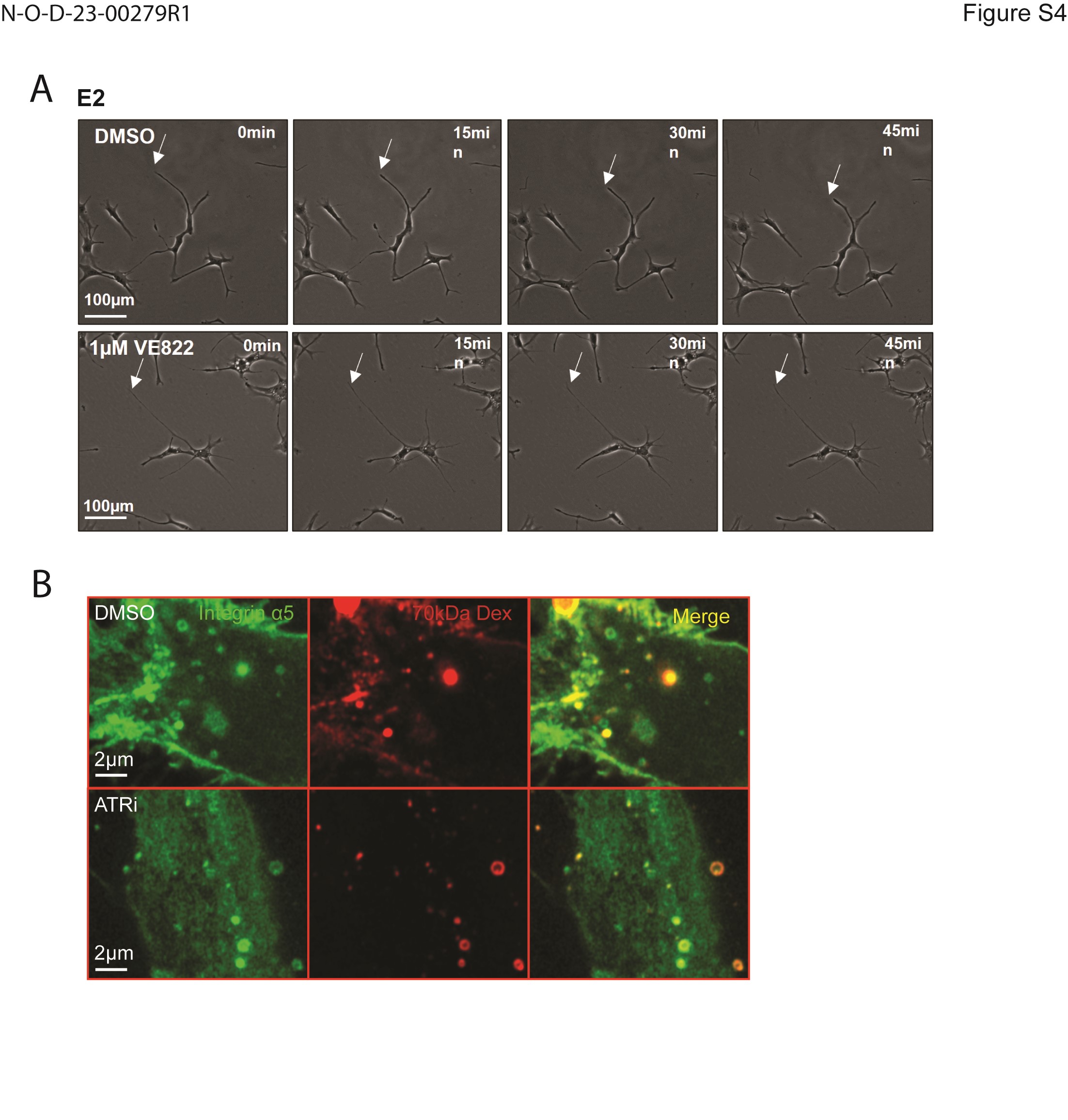

Supplement: noad210_suppl_Supplementary_Figure_S4 [file noad210_suppl_supplementary_figure_s4.jpeg]

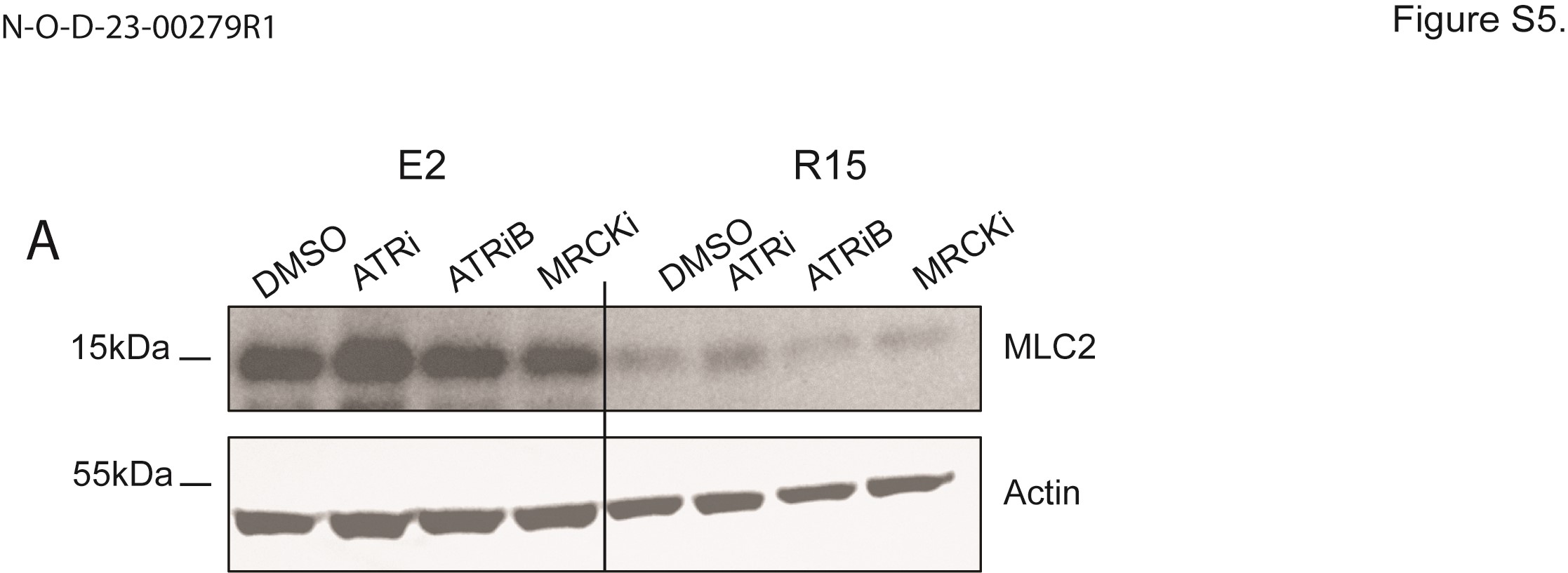

Supplement: noad210_suppl_Supplementary_Figure_S5 [file noad210_suppl_supplementary_figure_s5.jpeg]
