# Supplementary material for: Inhibition of ATR opposes glioblastoma invasion through disruption of cytoskeletal networks and integrin internalization via macropinocytosis
Source: Neuro Oncol. 2023 Nov 4;26(4):625–39. doi: 10.1093/neuonc/noad210 (PMC10995506; doi:10.1093/neuonc/noad210)
Supplement: noad210_suppl_Supplementary_Material [file noad210_suppl_supplementary_material.docx]

**Supplemental Material and Methods**

**CRISPR knock outs**

ATR CRISPR oligos were generated using ([https://www.zlab.bio/resources](https://eur03.safelinks.protection.outlook.com/?url=https%3A%2F%2Fwww.zlab.bio%2Fresources&data=05%7C01%7CJoanna.Birch%40glasgow.ac.uk%7Cca6fbd273f954d1becad08dbc6a3eca9%7C6e725c29763a4f5081f22e254f0133c8%7C1%7C0%7C638322179585670739%7CUnknown%7CTWFpbGZsb3d8eyJWIjoiMC4wLjAwMDAiLCJQIjoiV2luMzIiLCJBTiI6Ik1haWwiLCJXVCI6Mn0%3D%7C3000%7C%7C%7C&sdata=xig%2FnyocBYmAqTgcXo1CNsWwxsjTeEUR4uo7ti9slqQ%3D&reserved=0))  and ordered via the invitrogen oligo ordering tool. Guide plasmid plentiCRISPRv2 puro was transformed in Stbl3 cells at 30 degrees and packaging plasmids pVSV-G2 and psPAX2 were transformed in OneShot cells at 37 degrees. Vectors were digested, gel purified and the annealed and phosphorylated oligo pairs were ligated overnight at RT and transformed into Stbl3 cells at 30 degrees. Successful insertion of oligo sequence into plasmid was confirmed by Sanger sequencing of selected colonies. HEK293 cells were transfected with plasmids and virus harvested after 48 hours and added to cultures of G7 cells. Media was changed after 48 hours and cells selected under puromycin. hATR guide C was insufficient and used as a control guide. Sequences:

| hATR Guide C F | CACCGGAGCATGTGAAGTTACAATG |
| --- | --- |
| hATR Guide C R | AAACCATTGTAACTTCACATGCTCC |
|  |  |
| hATR Guide 1 F | CACCGTTGACATTACTGCAGTGGAA |
| hATR Guide 1 R | AAACTTCCACTGCAGTAATGTCAAC |
|  |  |
|  |  |
| hATR Guide 2 F | CACCGCTGACTTTGGTAGCATACAC |
| hATR Guide 2 R | AAACGTGTATGCTACCAAAGTCAGC |

**3D Scaffold invasion assay**

1 x10^4^ E2 or R15 cells/well were plated onto 96 well Alvetex scaffold plates (AmsBio, AMS.AVP009) that had been activated with 70% ethanol and coated with matrigel. These scaffold plate have previously been reported to indude a biologically relevant morphology in GBM cells [1]. Wells were treated with either DMSO, MRCKi (1 μM BDP9066, positive control), ATRi (1μM VE822) or ATRiB (1μM Bayer), 6 internal replicates per biological repeat. Cells were allowed to migrate for 5 (R15) or 7 (E2) days, before fixation with 4% paraformaldehyde and staining with HCS Cell Mask Deep Red Stain (ThermoFisher H32721). Cells that had reached the bottom 20μm of the scaffold were detected and counted using high-throughput imaging (Opera Phoenix) and automated analysis (Columbus).

**Autophagic flux assay**

Cells were plated at sub confluent density in 10cm petri dishes and incubated for 24 hours. DMSO, VE822 1µM and/or chloroquine 10µM were added to media and cells further incubated until lysis and harvesting at the appropriate timepoint. Cell lysates were subject to Western blotting with LC3B antibody (Cell Signalling 2775), as described previously [2]

**Transmission Electron microscopy**

Samples were fixed in 2.5% glutaraldehyde, 4% paraformaldehyde, in 0.1M cacodylate buffer, pH 7.2; washed in 0.1M cacodylate buffer, pH 7.2 and post-fixed in 1% OsO4 for 1 hour. After several washes in the same buffer, the samples were *en bloc* stained with 0.5% uranyl acetate in water for 30 minutes. Afterwards, samples were washed with water, dehydrated in ascending acetone series and resin embedded. Ultrathin sections (50nm thick) were collected and imaged on a JEOL 1200 Transmission electron microscope (JEOL, Japan) operating at 80kV.

**Derivation of primary GBM cell lines**

Primary GBM cell lines E2, G7, and R15 were derived from resected tumors and maintained as described previously [3] [4], approved by the local regional Ethics Committee (LREC ref 04/Q0108/60) in compliance with the UK Human Tissue Act 2004 (HTA License ref 12315). For OX5 cell line derivation, fresh treatment-naïve glioblastomas were collected from patients who provided informed consent undergoing surgery at Sheffield Teaching Hospitals NHS Foundation Trust (Ethical approval: Yorkshire & The Humber – Leeds East REC (11-YH-0319/STH15598)

1. Gomez-Roman, N., et al., *A novel 3D human glioblastoma cell culture system for modeling drug and radiation responses.* Neuro Oncol, 2017. **19**(2): p. 229-241.

2. Carruthers, R., et al., *Abrogation of radioresistance in glioblastoma stem-like cells by inhibition of ATM kinase.* Mol Oncol, 2015. **9**(1): p. 192-203.

3. Fael Al-Mayhani, T.M., et al., *An efficient method for derivation and propagation of glioblastoma cell lines that conserves the molecular profile of their original tumours.* J Neurosci Methods, 2009. **176**(2): p. 192-9.

4. Ahmed, S.U., et al., *Selective Inhibition of Parallel DNA Damage Response Pathways Optimizes Radiosensitization of Glioblastoma Stem-like Cells.* Cancer Res, 2015. **75**(20): p. 4416-28.
