# Supplementary material for: Inhibition of ATR opposes glioblastoma invasion through disruption of cytoskeletal networks and integrin internalization via macropinocytosis
Source: Neuro Oncol. 2023 Nov 4;26(4):625–39. doi: 10.1093/neuonc/noad210 (PMC10995506; doi:10.1093/neuonc/noad210)
Supplement: noad210_suppl_Supplementary_Table_S1 [file noad210_suppl_supplementary_table_s1.docx]

| **Cell line** | **0Gy Motility IC50** | **2Gy Motility IC50** | **0Gy Viability IC50** | **2Gy Viability IC50** |
| --- | --- | --- | --- | --- |
| **G7** | 0.15μM | 0.09μM | 7.7μM | 7.2μM |
| **E2** | 0.7μM | 0.41μM | 3.8μM | 3.9μM |
| **R15** | 0.23μM | 0.21μM | 3.9μM | 5.3μM |

**Table S1**
